# Effect of Structure on Charge Distribution in the Isatin Anions in Aprotic Environment: Spectral Study

**DOI:** 10.3390/molecules22111961

**Published:** 2017-11-14

**Authors:** Pavol Tisovský, Róbert Šandrik, Miroslav Horváth, Jana Donovalová, Juraj Filo, Martin Gáplovský, Klaudia Jakusová, Marek Cigáň, Róbert Sokolík, Anton Gáplovský

**Affiliations:** 1 Faculty of Natural Sciences, Institute of Chemistry, Comenius University, Ilkovičova 6, Mlynská dolina CH-2, SK-842 15 Bratislava, Slovakia; sandrik2@uniba.sk (R.Š.); mirek.horvath@gmail.com (M.H.); donovalova@fns.uniba.sk (J.D.); filo@fns.uniba.sk (J.F.); jakusova@fns.uniba.sk (K.J.); cigan@fns.uniba.sk (M.C.); sokolik@fns.uniba.sk (R.S.); gaplovsky@fns.uniba.sk (A.G.); 2 Department of Pharmaceutical Chemistry, Faculty of Pharmacy, Comenius University, Odbojárov 10, SK-832 32 Bratislava, Slovakia; m.gaplovsky@yahoo.com

**Keywords:** isatin azanions, azanion aggregation, counterion effect, UV–Vis, FTIR, NMR spectroscopy

## Abstract

Five isatin anions were prepared by deprotonation of initial isatins in aprotic solvents using basic fluoride and acetate anions (F^−^ and CH_3_COO^−^). The F^−^ basicity is sufficient to deprotonate isatin NH hydrogen from all the studied compounds. This process is reversible. In the presence of proton donor solvents, the anions form the corresponding isatins. The isatin hydrogen acidity depends on the overall structure of the isatin derivatives. The anions were characterized by ultraviolet–visible (UV–Vis), Fourier transform infrared (FTIR) and nuclear magnetic resonance (NMR) spectroscopy. Interestingly, the anions form aggregates at concentrations above 10^−3^ mol·dm^−3^. Further, the effect of cations on the UV–Vis spectra of the studied anions was studied. Charge transfer and its distribution in the anion depends on the radius and the cation electron configuration. The alkali metal cations, tetrabutylammonium (TBA^+^), Mg^2+^ and Ag^+^, interact with the C-2 carbonyl oxygen of the isatin anion. The interaction has a coulombic character. On the other hand, Cd^2+^, Zn^2+^, Hg^2+^, Co^2+^, and Cu^+^ cations form a coordinate bond with the isatin nitrogen.

## 1. Introduction

Isatin # and its derivatives belong to the most versatile organic compounds, especially in the field of practical applications. Their high application potential is mainly in areas like medicinal chemistry, such as antibiotic, antidepressant, anticancer, anti-human immunodeficiency virus (HIV), antimalarial and anti-tuberculosis drugs, and so on [1,2,3,4,5,6,7]; pesticides development; analytical reagents and dyes [8,9]; nanotechnologies, particularly the preparation of silver nanoparticles prepared in synergy with isatin derivatives [10,11]; novel heterocyclic compound synthesis and stereoselective processes [12,13,14,15,16,17,18,19,20,21]; organic material for electronics [22]; and polymerization [23].

The high application potency of isatin and its derivatives, their occurrence, and their metabolites’ occurrence in plants and in the human body has prompted great interest from chemists, physicians, and pharmacists to study their chemical reactivity. This issue has been, and is still, devoted a great deal of attention, in terms of preparing new derivatives as well as studying the mechanisms of their transformation in plants and in various animal organisms. Due to these facts and also due to the chemical structure itself, increased attention has been paid to the kinetics of isatin hydrolysis, and the respective derivatives [24,25,26,27,28,29,30,31,32]. From these works follows that the rate of hydrolysis does not show a simple dependence on the environment pH. There are changes in mechanism and the rate-determining step, and the hydrolysis mechanism depends on the environment polarity. The hydrolysis of isatin and its derivatives is a complex process in which, depending on the conditions, several reaction steps or intermediates compete with each other. One of the intermediates predicted by some authors [26] is the conjugated isatin anion (III). The physicochemical anion properties differ from the neutral molecule physicochemical properties. Anions, due to weaker bonded valence electrons, make stronger van der Waals interactions with surrounding molecules than more compact and less polarizable neutral molecules. These interactions can also significantly affect the anion reactivities. The imide and amide anion nucleophilic reactivity was tested by the reaction with various electrophilic substrates [33]. Bunnett and Beale studied the reaction kinetics of several imide and sulfonamide anions with methyl iodide and methyl methanesulfonate in methanol. They reported that the nucleophilic reactivity of these anions correlates with their basicity [34,35]. The ease of anion formation, their stability or reactivity, and likely the biological activity, depend on the anion structure, environment, and so on. Even though the isatin anion or its structural modification may play an important role in the metabolism process of isatin and its derivatives, not enough attention has been paid to the study of their properties so far. Fourier transform infrared (FTIR) and Raman spectra of the isatin anion itself were published by Binev et al. [36].

The photophysics of the isatin anion were described in the work of Berci-Filho et al. [37]. As mentioned above, isatin has high application potential in various areas of industry. An isatin structural fragment is often a part of functional materials. The isatin structure is found in several compounds whose properties have been designed in the area of chemical anion sensors [38,39], or materials usable in electronics [40]. For this area of functional material applications, we have recently used the isatin fragment as the carrier structure in the development of anion sensors, or signal switches. The isatin fragment, depending on the overall structure of the molecule, can enter into tautomeric equilibria that affect the entire molecule functionality. When designing structures of such functional materials, it is necessary to know the spectral, physical, photophysical, and chemical properties of the molecules with the isatin structure as well as their azanions. Therefore, in this work, we have focused on the preparation and spectral properties study (ultraviolet–visible (UV–Vis), FTIR, nuclear magnetic resonance (NMR) of isatin azanions depending on their structure.

Organic anions can be generated in various ways. For compounds that have acidic hydrogen in their molecule, the following reaction is often used to generate anions:

X^−^ + H–Y ↔ H–X + Y^−^. (1)

The H–Y strong acid anion can be prepared by reaction with an anion X^−^ and a weaker H–X acid. If the anion X-basicity is insufficient to deprotonate the HY receptor, a hydrogen bond is formed between HY and X:

X + H–Y ↔ Y–HX^−^ (2)

**Scheme 1:**
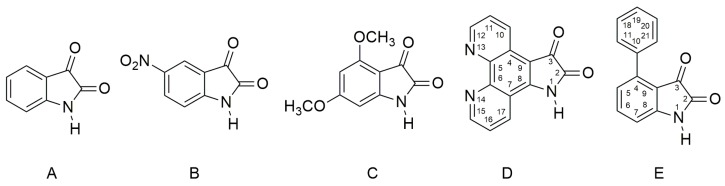
Molecular structures of studied isatin derivatives **A**–**E**. Isatin **D** is a new compound, and so far, it has not yet been described in literature.

There are many anion examples—F^−^, AcO^−^ and so on—which interact with the acidic hydrogen of an urea fragment, or with the hydrogen of *N*,*N′*-bis(diphenyl)urea derivatives and the NH hydrogen of acidic benzimidazoles, pyrroles, and indoles to form the corresponding anions [41,42,43,44,45,46,47,48,49,50,51]. In this work we used the following isatins (Scheme 1) to prepare their azanions and to study the effect of their structure on spectral properties (UV–Vis, FTIR and NMR).

## 2. Results and Discussion

### 2.1. Ultraviolet–Visible Spectra of the Compounds ***A***–***E*** and Their Azanions

**Scheme 2:**
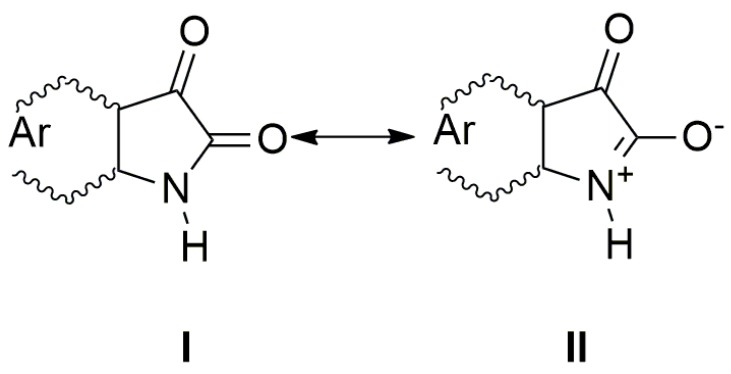
The charge distribution in the studied molecules.

**Figure 1:**
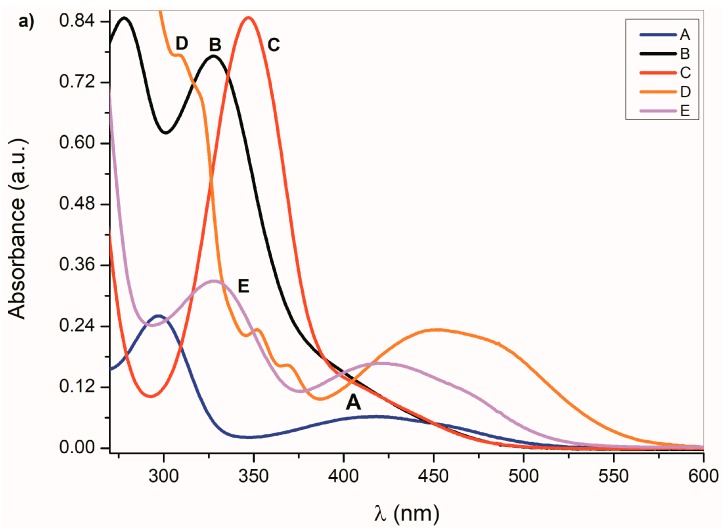

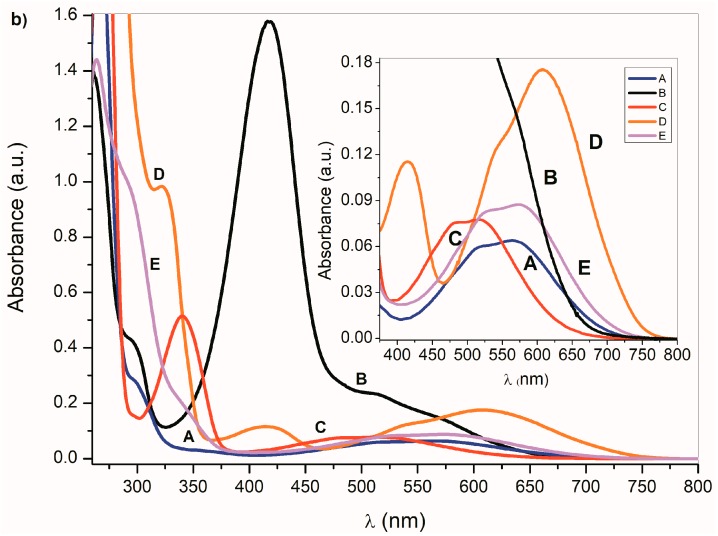
(**a**) Ultraviolet–visible (UV–Vis) spectra **A**–**E** (1 × 10^−4^ mol·dm^−3^) and (**b**) corresponding azanions in the presence of tetrabutylammonium fluoride (TBAF) in dimethyl sulfoxide (DMSO) (1 × 10^−2^ mol·dm^−3^).

The UV–Vis spectra of **A**–**E** and the spectra of their corresponding azanions are shown in Figure 1a,b. Absorption maxima in the region 260 nm to 350 nm correspond to the π→π* transition of the aromatic part of the structure **A**–**E** [52]. A relatively weak absorption band ranging from 350 nm to 600 nm is linked with the nitrogen and oxygen free electron pairs and can be assigned to n→π* and intramolecular charge transfer (ICT) transition. The charge distribution in the molecule can be described by the resonance structure that is characteristic for amides or lactams (Scheme 2). The nitrogen free electron pair delocalization supports the formation of a partial double bond between nitrogen and the isatin C-2 position (structure II, Scheme 2).

Any change that directly affects the electron density on the nitrogen atom will also affect the physicochemical properties of the studied compounds. The absorption maxima position as well as the band intensity from 260 to 350 nm therefore depends on the structure or the donor–acceptor ability of the studied isatin aromatic systems. The structural comparison of **A**, **C** and **E** shows that the position of these bands shifts bathochromically with increasing donor ability of the **A**, **C** and **E** aromatic system (Figure 1a). Similarly, the intensity of these absorption bands is proportional to the donor strength of the aromatic system of the isatins.

Such dependence of the absorption intensity on the isatin aromatic system donor strength was not observed for the long-wavelength absorption in the 350 nm to 600 nm region. The π→π* and n→π* dependence on the donor strength of the **A**–**E** aromatic system indicates that the interaction of the aromatic ring and the isatin five-membered ring cannot be described by a one-parameter function, but it is, rather, a complex process [53]. Erich Kleinpeter et al. [54] showed that through space nuclear magnetic resonance shieldings (TSNMRS) can be successfully used to quantify and visualize (anti-)aromaticity and to easily identify the zwitterionic structure in push–pull systems. From theoretical calculations and the results of the experimental measurements, they show that the isatin five-member ring has an anti-aromatic character, contrary to its six-member ring. Therefore, we assume that the above-mentioned **A**–**E** UV–Vis spectral changes depend on the relative aromaticity/anti-aromaticity differences of both structural fragments and the related degree of zwitterionic structure in the molecule.

**Scheme 3:**
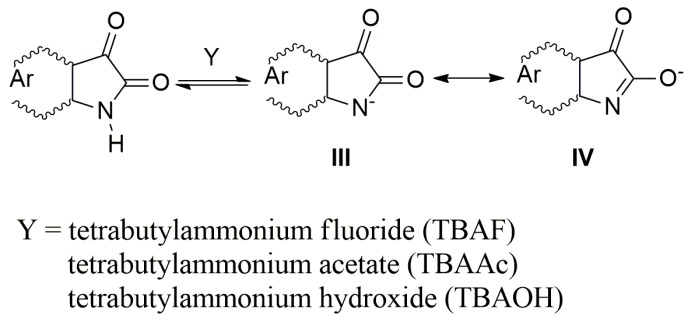
Resonance structure of the studied azanions (**A**–**E**).

In the presence of a base, the NH hydrogen deprotonation from the **A**–**E** nitrogen occurs and the corresponding azanion III is formed (Scheme 3). The long-wavelength **A**–**E** absorption bands in the 350–600 nm region (Figure 1a) disappear and new azanion bathochromic shifted bands appear in the 400 nm to 750 nm region (Figure 1b). 

Compared with the **A**–**E** long-wavelength absorption maxima, the corresponding azanions’ absorption maxima are significantly red-shifted (approximately 100 nm) (Table 1). This shift results from the delocalization of the nitrogen free electron pairs on the –N–C=O fragment of the azanion five-membered ring (resonance structures III and IV—Scheme 3), including delocalization towards the aromatic six-membered phenyl ring (which will be discussed further).

The long-wavelength absorption bands batochromic shift (n→π* and ICT) of the **A**–**E** azanions depends on the structure of the parent compounds (**A**–**E**). The n→π*/ICT absorption bands of **A**, **C**, **D** and **E**, are well separated from the corresponding π→π* bands. However, the **B**-azanion long-wavelength n→π* absorption band and its relatively intense π→π* absorption at 417 nm overlap.

**Figure 2:**
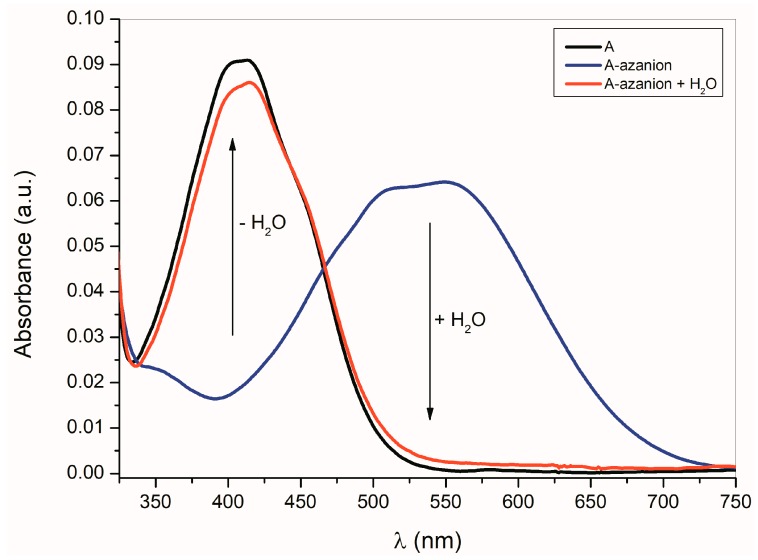
Effect of water (c_H2O_ = 1 × 1.85 mol·dm^−3^) on **A**-azanion UV–Vis spectrum in acetonitrile, (c_A_ = 1 × 10^−4^ mol·dm^−3^; c_TBAF_ = 1 × 10^−3^ mol·dm^−3^).

As shown in Equation (1), the formation of **A**–**E** azanions is a reversible process. The protonodonor solvent (e.g., water, methanol, etc.) addition to the azanion solutions in dimethyl sulfoxide (DMSO), dimethylformamide (DMF) or CH_3_CN results in the initialization of isatin **A**–**E** recovery (Figure 2).

The UV–Vis spectra of azanions are also affected by their concentration. The tetrabutylammonium fluoride addition (TBAF; 5 × 10^−1^ mol·dm^−3^) to the concentrated (1 × 10^−2^ mol·dm^−3^) **A** solution leads to the appearance of a new bathochromically shifted (≈67 nm) absorption band at 479 nm (Supplementary Figure S1). The 10-times dilution of this solution results in the initial 565 nm shoulder band intensity increase and, thus, suggests the intermolecular process. We assume that the **A**-azanion undergoes aggregation. The absorption maximum at 479 nm was assigned to the aggregated form of the azanion of **A**. The **B**-azanion concentration effect on its UV–Vis spectrum is shown in Supplementary Figure S2. The **B**-azanion long-wavelength band intensity at 410 nm increases with decreasing azanion concentration and its maximum position is hypsochromically shifted by 4 nm. Concentration changes in the UV–Vis spectra of other studied (**C**–**E**) azanions lie between these boundary examples (between Supplementary Figures S1 and S2) and depend on the overall anion structure/substituent effect.

### 2.2. The Influence of the Acid Anions (F^−^, CH_3_COO^−^, Cl^−^, Br^−^, NO_3_^−^, HSO_4_^−^) and Counter Ions on the Ultraviolet–Visible Spectra of ***A***–***E*** and Their Azanions

Experimental results show that the F**^−^** (TBAF) anion basicity is sufficient to remove the isatin NH proton from all the studied compounds (**A**–**E**) in aprotic solvents. The deprotonation process selectivity increases with the decrease of the anion basicity: for example, if CH_3_COO^−^ (tetrabutylammonium acetate, TBAAc) is used instead of the F^−^ anion. Contrary to the strongly basic F^−^ anion, the addition of the less-basic CH_3_COO^−^ anion to the solution of isatin **C** with two strong electrodonor methoxy groups in its structure does not lead to NH hydrogen deprotonation even at high CH_3_COO^−^ concentration (1 × 10^−2^ mol·dm^−3^; ESI Figure S3). Because the **A** and **C** isatin NH hydrogen acidities are similar, the observed changes in the UV–Vis spectra are negligible compared with those observed after the F^−^ anion addition (Supplementary Figure S3). However, the electron-acceptor properties of the nitro group in the 5-position of **B** and the two C=N groups in **D** increase the acidity of the NH hydrogen and, therefore, CH_3_COO^−^ basicity is sufficient to generate the corresponding azanions (Supplementary Figure S4). Less basic Cl^−^, Br^−^, NO_3_^−^, or HSO_4_^−^ anions are not able to deprotonate isatins **A**–**E**. The azanions generated by **A**–**E** deprotonation in DMSO, DMF, CH_3_CN, and CHCl_3_ are stable even at a high concentration (1 × 10^−2^ mol·dm^−3^) of F^−^ or CH_3_COO^−^ at room temperature, and their addition does not result in the isatin ring opening.

**Scheme 4:**
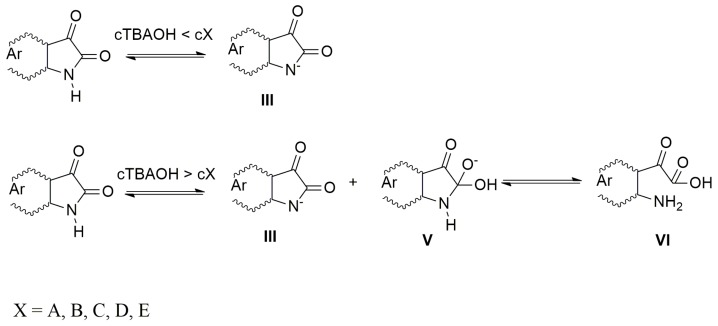
Mechanism of azanion formation.

When the azanions are generated with the tetrabutylammonium hydroxide (TBAOH), the azanion stability strongly depends on the base concentration. At TBAOH concentrations lower than 1 equiv of the corresponding isatins (c_TBAOH_ < c_X_, X = **A**–**E**), the UV–Vis spectra of generated azanions are not time-dependent and they are the same as the UV–Vis spectra of azanions generated by TBAF (Supplementary Figure S5). At a higher TBAOH concentration (c_TBAOH_ > c_X_, X = **A**–**E**), the **A**–**E** azanions long-wavelength band intensity decreases over time (Supplementary Figure S5) with a simultaneous absorption increase in the 350–475 nm region. The newly emerging band is characteristic for the isatin open form (2-(2-aminophenyl)-2-oxoacetic acid) and its presence was confirmed by the synthetized 2-(2-aminophenyl)-2-oxoacetic acid TBA salt 2-(2-aminophenyl)-2-oxoacetic acid (reaction of 2-(2-aminophenyl)-2-oxoacetic acid with TBAOH). The UV–Vis spectrum of TBA salt 2-(2-aminophenyl)-2-oxoacetic acid is identical to the UV–Vis spectrum of **B** after TBAOH addition (1 × 10^−2^ mol·dm^−3^) (Supplementary Figure S6). At low OH^−^ concentration (c_TBAOH_ < c_X_, X = **A**–**E**), the hydroxide ions are preferably consumed in the deprotonation process to form the azanion III (Scheme 4).

The rate of C-2 nucleophilic attack by OH^−^ cannot compete with the acid–base reaction rate under these conditions. At high TBAOH concentration (c_TBAOH_ > c_X_, X = **A**–**E**), not all OH^−^ ions are consumed to deprotonate these compounds and free OH^−^ ions nucleophilically attack the **A**–**E** C-2 carbon. The five-member ring of the intermediate V rapidly opens to form VI (2-(2-aminophenyl)-2-oxoacetic acid) in the next reaction step. Supplementary Figure S7 shows the effect of water on the UV–Vis spectrum of **B** in the presence of TBAOH. Immediately after TBAOH addition to **B** in DMSO, the spectrum of B (spectrum 1; Supplementary Figure S7) shows two maxima at 420 and 470 nm corresponding to forms III and VI. The VI concentration increases with time (spectrum 2, Supplementary Figure S7). The addition of water shifts the reaction equilibrium backwards (spectrum 3, Supplementary Figure S7). The absence of intermediate V formation in the UV–Vis spectra during the back reaction indicates that the rate-determining step in this chemical transformation is the isatin C-2 nucleophilic attack (Scheme 4). The five-member ring opening is a rapid process. The OH^−^ attack of dimethoxy isatin **C** does not provide the products V nor VI even at a high TBAOH concentration of 1 × 10^−2^ mol·dm^−3^. The isatin **C** is stable under these conditions and, therefore, its UV–Vis spectrum does not change due to the C-2 electrophilicity decrease resulting from the donor (+M) effect of the methoxy groups. The VI formation-rate constants are shown in Table 2.

The experiments with *N*-methylated derivatives **A**–**E** (with isatin NH hydrogen structural fragment replaced by the methyl group) support the previously mentioned results. Compared with **A**–**E** (Scheme 4), the entire reaction process is simplified in the methylated compounds. These compounds are not able to form azanions III in the presence of bases. The presence of TBAF (TBAOH) results in the C-2 nucleophilic attack by F^−^ (OH^−^) and the subsequent ring opening in an aprotic environment [55]. The effect of the water presence on the methylated derivatives can be seen in ESI Figure S8. The equilibrium state is reached immediately after the water addition. Similarly to **A**–**E**, the reactivity of the methylated analogs is influenced by their structure and the base strength. The increase in absorbance corresponds to the reaction product formation, i.e., the open form of the methylated compounds. As expected, the intermediate formation was not observed in UV–Vis spectra. Similarly to unmethylated **B**, the 1-methyl-5-nitroisatin has a clearly visible band with a peak around 490 nm (Supplementary Figure S8) in the presence of the base (TBAF, TBAOH), which we have assigned to an intermediate formed by C-2 nucleophilic attack with the base.

**Figure 3:**
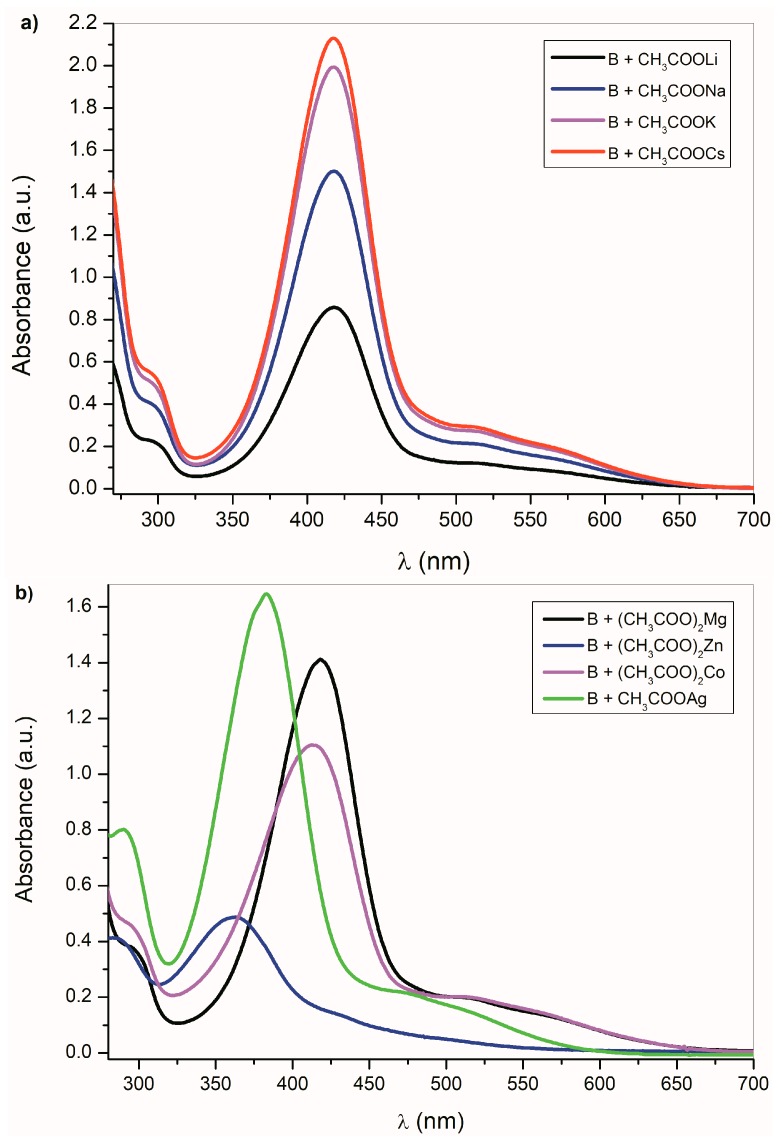
**B** UV–Vis spectra change (1 × 10^−4^ mol·dm^−3^) in the presence of (**a**) alkali metal acetates (**b**) other metal acetates in DMSO.

The UV–Vis spectra of **A**–**E** anions are also dependent on the counter ion. The **A**–**E** anions with different cations can be prepared by direct reaction of **A**–**E** anions (generated by the reaction with F^−^ or CH_3_COO^−^) with corresponding acetates or other metal salts (Figure 3; Supplementary Figures S9 and S10).

This process is universal and applicable to all studied compounds. As expected, the UV–Vis spectral changes of **A**–**E** are dependent on the cation type. Figure 3a shows the UV–Vis spectra of **B** in the presence of alkali metal acetates. As was discussed above, the CH_3_COO^−^ ions are able to deprotonate the studied compounds **A**–**E**, except the isatin **C**. Although the absorption bands position of **B**-azanion with the corresponding alkali metal cation (Figure 3a) do not change, the absorption intensity of the entire spectrum varies with the cation type. Due to the change in equilibrium between **B** and its azanion and/or the **B**-azanion aggregation degree, the absorption intensity change in the UV–Vis spectrum depends on the cation ionic radius. The Mg^2+^ cation has the same effect on the spectrum. The effect of Ag^+^, Cd^2+^, Zn^2+^, Hg^2+^, Co^2+^, and Cu^+^ on the UV–Vis spectrum of **B** in the presence of TBAF is showed in Figure 3b and ESI Figure S11. Contrary to alkali metal cations (Figure 3a), a significant hypsochromic shift of the absorption bands can be observed in the spectrum (Figure 3b and Supplementary Figure S11). We assume that this behavior reflects the different mechanism of the cation interaction with the corresponding anion. Alkali metal, TBA^+^, and Mg^2+^ cations have no free electrons in the valence sphere, are poorly polarizable, have relatively high charge density and hard acid properties, and their anion binding is therefore predominantly coulombic. On the other hand, Cd^2+^, Zn^2+^, Hg^2+^, Co^2+^, and Cu^+^ cations form a coordinate bond with the isatin nitrogen.

### 2.3. The Azanions Fourier Transform Infrared Study

Vibration spectra of substituted isatins were studied earlier [56,57,58]. The main frequency assignment to the corresponding vibration mode has been accomplished based on the IR and Raman spectra and single crystal dichroism [56]. Fourier transform infrared spectra of **A**–**E** are shown in Supplementary Figure S12. Characteristic vibrations of the isatin five-membered ring are effected by the induction and resonance effects of the aromatic structural fragment in these molecules [59]. The carbonyl stretching vibrations of C=O lie in 1850–1600 cm^−1^ region [60]. The two carbonyl bands are observed in the FTIR spectrum. The band at the higher frequency corresponds to *ν_as_(C=O)* and the band at the lower frequency is assigned to *ν_s_(C=O)*. Resolution of the two bands depends on the compound structure (Supplementary Figure S12) and the environment. Bands corresponding to the C–N stretching vibrations (1200–1400 cm^−1^) are relatively intense and mostly overlap with C–C and C–C–H bands [61,62].

**Figure 4:**
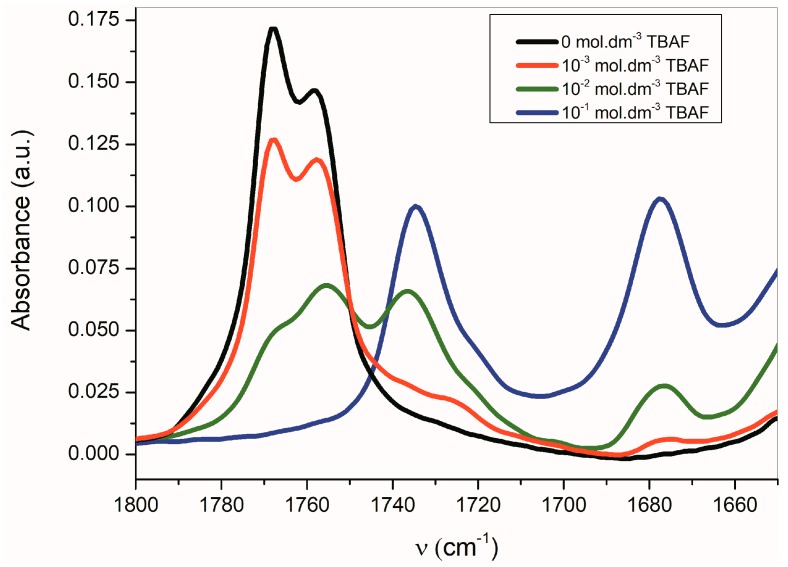
Change of the Fourier transform infrared (FTIR) spectra of **B** (1 × 10^−2^ mol·dm^−3^) in CH_3_CN depending on concentration of TBAF.

Similarly to the UV–Vis spectra, the **A**–**E** azanions FTIR spectra differ from the parent isatin **A**–**E** FTIR spectra (Supplementary Figure S12). These changes are observable particularly in the carbonyl region (Table 3). Once the azanion has been formed, both carbonyls are shifted to lower frequencies. Due to the strong resonance between the α-C=O and the azanion center, the azanions’ FTIR spectra show a significant reduction in the frequency of the corresponding α-C=O. Transformation to the corresponding azanion can be seen in the titration experiment with TBAF (Figure 4).

The band corresponding to *ν_as_(α-C=O)* is more sensitive to the presence of TBAF than the *ν_s_(β-C=O)* band. The disappearance of the *ν_as_(α-C=O)* band with the maximum at 1768 cm^−1^ occurs relatively quickly, while the *ν_s_(β-C=O)* band intensity at 1758 cm^−1^ decreases more slowly. Simultaneously, the small shift of the maximum to the lower frequency occurs. At the TBAF concentration of 1 × 10^−2^ mol·dm^−3^ (1 equiv), four observable bands are present in the FTIR spectrum (with the maxima at 1768, 1755, 1735, and 1677 cm^−1^; Figure 4). At the TBAF concentration of 1 × 10^−1^ mol·dm^−3^ (10 equiv), only two maxima at 1735 and 1677 cm^−1^, with approximately the same intensity, are observed. Particularly, an uneven change of the *ν_as_(α-C=O)* and *ν_s_(β-C=O)* band intensities with the TBAF concentration change indicates the presence of another equilibrium (aside from the equilibrium between **B** and its azanion) (Supplementary Figure S2).

**Scheme 5:**
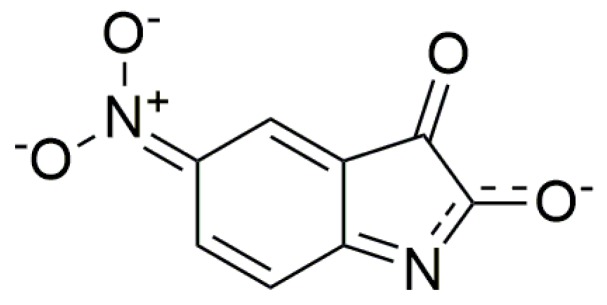
Resonance structure of the **B**-azanion.

In addition to changes in the **B**-azanion carbonyl region measured in the solid state—attenuated total reflection (ATR technique), large changes in the 1600–1500 cm^−1^ and 1330–1100 cm^−1^ region are notable (Supplementary Figure S12). Bands with the maximum at 1533 cm^−1^ or 1332 cm^−1^, which correspond to *ν_as_(C–N)* and *ν_s_(C–N)* of the nitro group of **B**, are shifted to the lower frequencies for the corresponding azanion (*ν_as_(C–N)* at 1487 cm^−1^ and *ν_s_(C–N)* at 1321 cm^−1^). The C–N stretching vibration of **B** (852 cm^−1^), characteristic for the nitro group, was shifted about 4 cm^−1^ to a lower frequency in the **B**-azanion. Big changes in the **B**-azanion FTIR spectrum were also observed in the plane-bending C–H vibration of the ring (in the region of 1300 to 1000 cm^−1^). There are several new strong and relatively wide bands in this area, e.g., at 1263 cm^−1^, 1104 cm^−1^. These changes, along with the intensity changes at about 1600 cm^−1^ (probably *ν(C=N)*), point to the different charge distribution of the **B**-azanion and can be described by the resonance structure (Scheme 5) [63,64].

**Figure 5:**
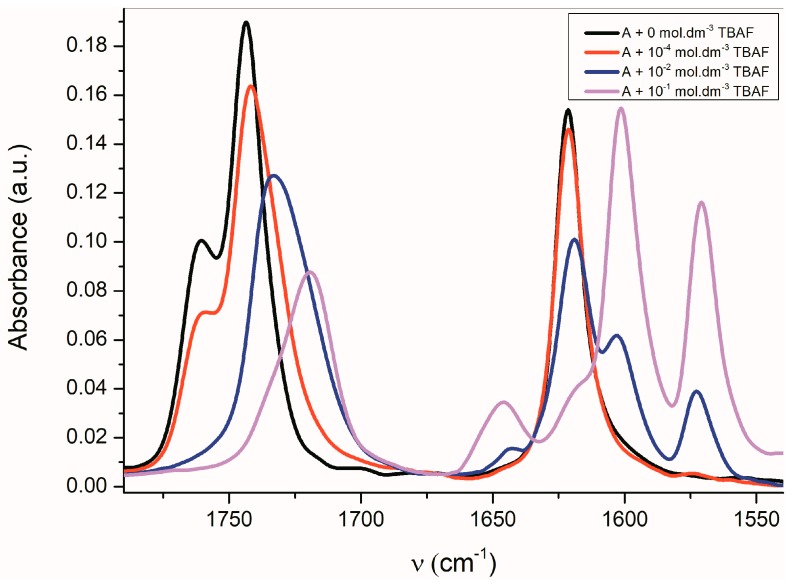
Change of FTIR spectra of **A** (1 × 10^−2^ mol·dm^−3^) in CHCl_3_ depending on the TBAF concentration.

The FTIR spectra of all studied **A**–**E** azanions are also significantly changed in the region of skeletal vibration (carbon–carbon stretching vibration in the phenyl ring and skeletal vibrations involving carbon–carbon stretching within the ring absorb in the 1620–1560 cm^−1^ and in the 1500–1400 cm^−1^ regions) (Figure 5 and Supplementary Figure S12), especially the characteristic doublet at 1600 cm^−1^ and 1570 cm^−1^.

**Scheme 6:**
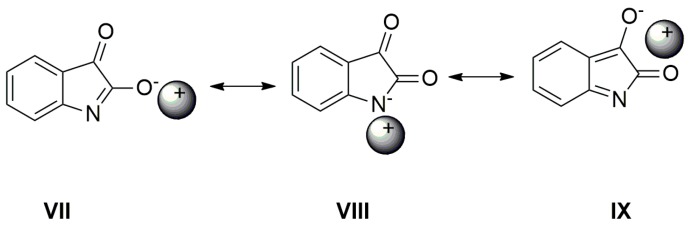
Cation can affect the charge distribution in the isatin anion.

The position of these bands depends only little on the solvent type or on the isatin sample phase (solution vs. solid state). The FTIR spectra indicate that the azanion negative charge delocalization depends on the structure of the corresponding compounds **A**–**E**. As shown in the UV–Vis spectra, the charge transfer, or its distribution in the molecule, depends on the cation type. The following resonance structures suggest that the cation can affect the charge distribution in the isatin anion (Scheme 6).

**Figure 6:**
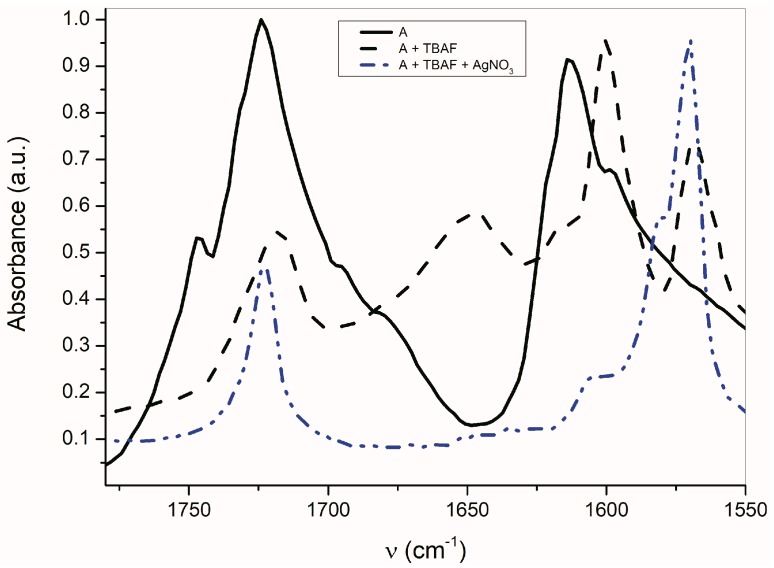
Effect of TBA^+^ and Ag^+^ on **A**-azanion FTIR spectrum (ATR spectra).

As seen in Figure 6, the AgNO_3_ addition to the mixture of **A** with TBAF (anion VII) significantly affects the FTIR spectrum. The *ν_as_(α-C=O)* band completely disappears and the second carbonyl half-width narrows, together with a simultaneous shift of its maximum to a higher frequency (4 cm^−1^).

**Figure 7:**
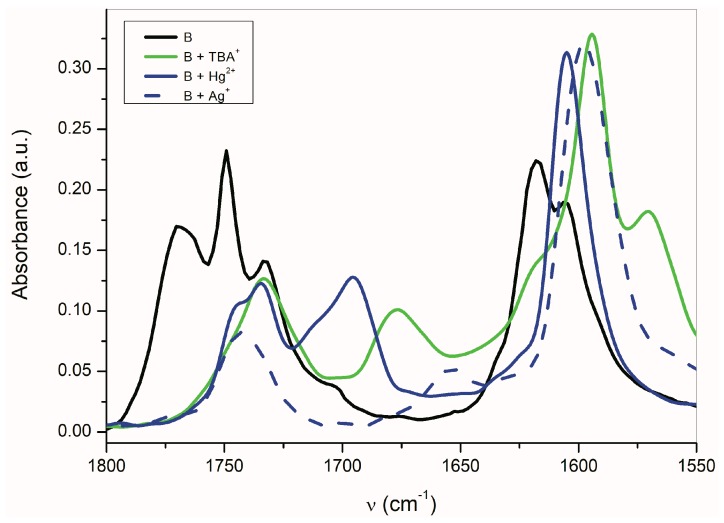
Effect of the counter ion on the **B**-azanion FTIR spectrum (ATR).

The band at 1614 cm^−1^ also disappears and the FTIR spectrum is identical to the silver salt of **A** (Supplementary Figure S13), which was isolated in pure form. As mentioned above in the discussion related to UV–Vis absorption, metal cations (Ag^+^, Cd^2+^, Zn^2+^, Hg^2+^, Co^2+^ and Cu^+^) can form a coordinate bond with the **A**-anion nitrogen. Similarly to the UV–Vis spectra, the addition of AgNO_3_ to **A** does not change the FTIR spectrum of **A**. After coordinate bond formation, the bands at 1645 and 1600 cm^−1^ disappear. These bands are characteristic for the studied azanions with the TBA^+^ or alkali metal counter ion (VII, Scheme 6). The disappearance of these bands (*ν_as_(C=O)*, *ν(C=N)*) indicates the charge distribution reorganization in the azanion molecule depicted in structure VIII (Scheme 6). This charge reorganization in the **A**–**E** anions is markedly reflected in the carbonyl vibrational region (Figure 7), particularly in the carbonyl stretching vibrations of azanion *ν_as_(α-C=O)*. The position of this band, depending on the counter ion, is shifted to lower frequencies as follows: Ag^+^ < TBA^+^ < Hg^2+^.

The shift of the carbonyl band to a lower frequency indicates that the C=O double bond has more single bond character, e.g., the greater part of the negative charge of the nitrogen azanion is located on the carbonyl oxygen. The transfer of the entire charge to the carbonyl oxygen of the C=O double bond leads to the enolate formation. Based on the *ν_as_(α-C=O)* band position, the coulombic interaction of carbonyl oxygen with the counter ion decreases in the following order: Ag^+^ > TBA^+^ > Hg^2+^. The silver cation leads to a structure very similar to the enolate (=C–O^−^Ag^+^) form. In the case of Hg^2+^ cation, the character of the carbonyl is retained, and this counter ion interacts primarily with the isatin nitrogen. This conclusion is consistent with the authors of references [65,66,67,68], who studied the alkylation regioselectivity of amides and lactams. The existence of the coordinate bond between the isatin **A** anion nitrogen and Hg^2+^ was also confirmed by X-ray spectroscopy [69]. The two deprotonated non-coplanar isatin ligands are in *trans* position to each other and are bound to Hg^2+^ in a head-to-tail orientation via the heterocyclic nitrogen in a linear N–Hg–N arrangement [69].

**Figure 8:**
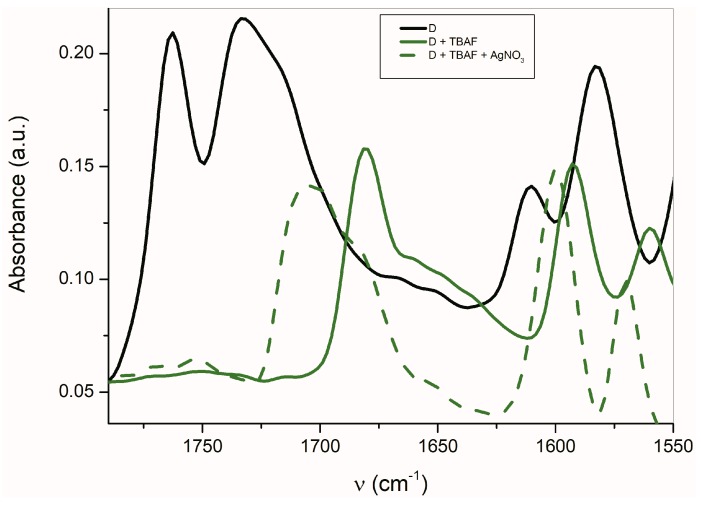
FTIR spectra of **D** in the presence of TBA^+^ and Ag^+^ (ATR spectra).

The FTIR carbonyl signal is shifted to higher frequencies (about 24 cm^−1^) for **D** in the presence of Ag^+^ compared with TBA^+^ (Figure 8). Similarly, the band observed at 1592 cm^−1^ is also shifted to a higher frequency (8 cm^−1^). This behavior is opposite to that of other studied azanions. Contrary to other isatins, the described changes in band shift indicate the Ag^+^ interaction with the nitrogens of the phenanthroline skeleton which result in the *ν(C=C)* shift to a higher frequency [70].

### 2.4. ^1^H and ^13^C Nuclear Magnetic Resonance Study

The amide nitrogen free electron pair delocalization formed by the isatin NH nitrogen deprotonation in the studied **A**–**E** azanions, leads to the chemical shift decreases in all of **A**, **B**, **D**, **E** azanion’s protons in the ^1^H-NMR spectrum, except the proton signals for the **C**-azanion (Table 4). C4, C5, and C6 chemical shift decrease (upfield shift of the corresponding carbon signals) is characteristic for **A**, **C**–**E** azanions (except the C6 **B**- and **D**-azanion signal) in ^13^C-NMR spectra compared to initial isatins.

The most significant change in ^13^C-NMR occurs on carbon C8. The chemical shift increase of more than 20 ppm observed for **B** and **D** azanions may indicate the C8 transformation from aromatic to the quasi–C=N bond character and, thus, the formation of the quinoid structure (Scheme 5). Such a structure is stabilized by the presence of a nitro group in para position towards C8 of **B** and phenanthroline nitrogen of **D**. These conclusions are supported by previously mentioned FTIR measurements.

The C2, C3, C7 and C9 carbon shifts of **A**–**E** azanions are higher than those of the carbons in the corresponding **A**–**E** isatin derivatives. The highest NH hydrogen chemical shift was measured for the isatin derivative **D** (Table 5). Experimentally detected chemical shifts of the N–H proton correlate with isatin p*K*_a_ values (Table 6) for the corresponding isatins, except for the isatin **D**. Results from NMR measurements are consistent with experimental results obtained by UV–Vis and FTIR spectroscopy.

### 2.5. Quantum-Chemical Calculation

**Figure 9:**
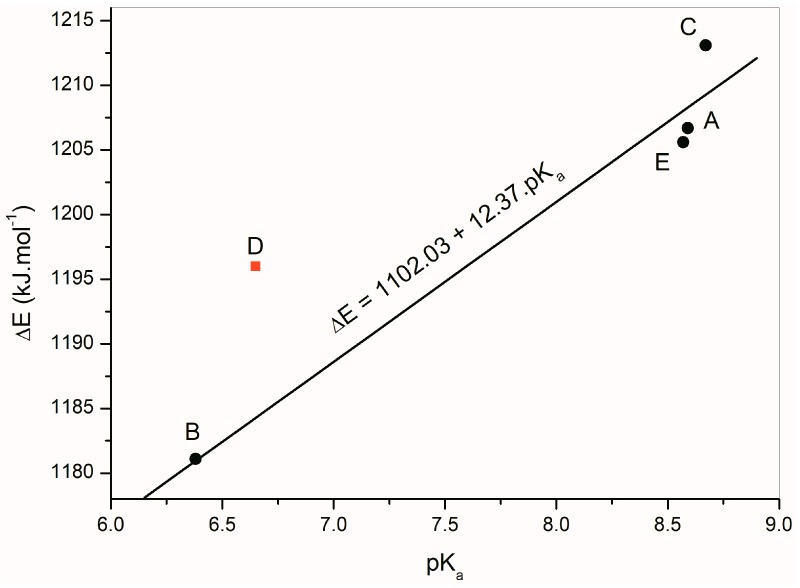
Linear dependence of the calculated deprotonation energies of **A**–**E** determined from experimental p*K*_a_ values of isatin derivatives.

The deprotonation energies of the studied isatin derivatives are listed in Table 6. These energy values determining the NH hydrogen acidic or basic properties are influenced mainly by the aromatic part of the individual structures. The p*K*_a_ values of the studied compounds were determined experimentally (Table 6). Figure 9 depicts a linear dependence of the calculated deprotonation energies of **A**–**E** on their experimentally measured p*K*_a_ values. The linear regression equation ΔE = 1102.03 + 12.37 p*K*_a_, (correlation coefficient *R* = 0.9821) was found for isatins **A**, **B**, **C** and **E**. Isatin **D** was excluded from the calculation because its deprotonation energy does not correlate with the rest of the compounds.

**Figure 10:**
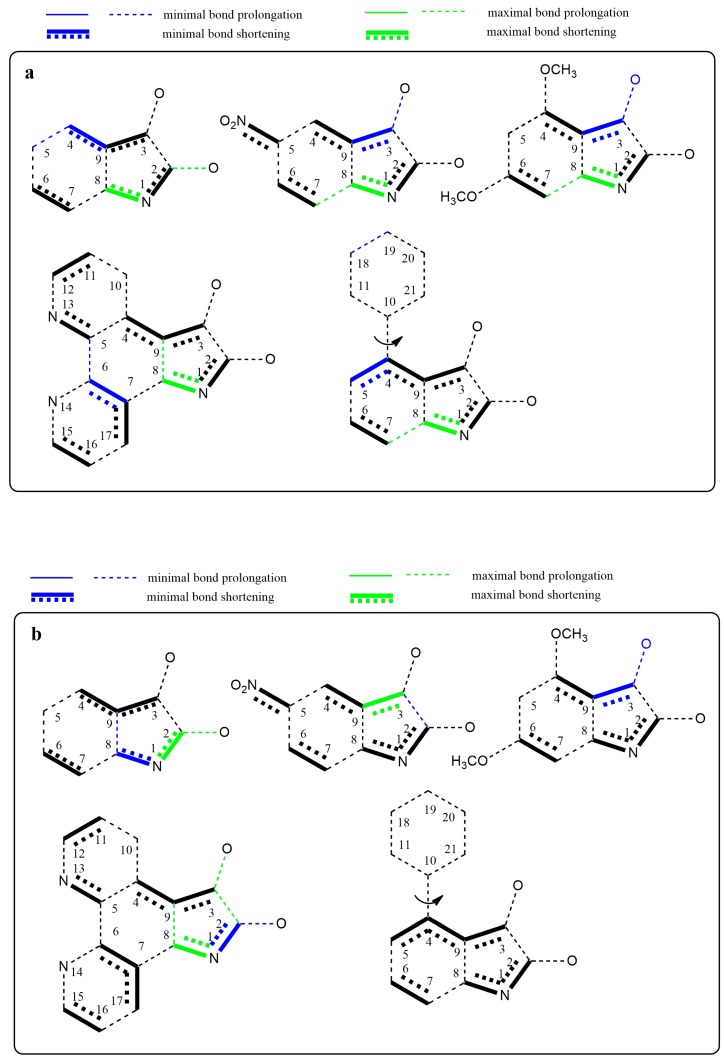
Graphical representation of the bond length differences. (**a**) Comparison of the isatin derivatives and their respective azanions; (**b**) depiction of the most and least distinct bond length changes caused by deprotonation of the isatine derivatives **A**–**E**.

Changes in the electron structure resulting from the conversion of studied isatins to their azanions are demonstrated by changes in the bond lengths and the partial charge of the atoms (Supplementary Tables S1 and S2). For the isatin and its substituted derivatives, the longer covalent bond between carbonyls is characteristic compared with other α-diketones [71,72]. Our calculations show that this bond length is even more prolonged in the studied azanions, mainly due to the increased charge delocalization (Supplementary Table S1). Moreover, the length of both carbonyl bonds is prolonged. This is consistent with the carbonyl band shift to the lower frequencies in FTIR spectra compared to the neutral isatins. Since all bonds of the –CO–CO– fragment of azanions are longer than those in the neutral structures, the mutual electron conjugation of carbonyls is reduced in the anionic structures. However, the two carbonyl groups are in conjugation with the aromatic part of the molecule. The bond length differences in isatins and their corresponding azanions are depicted in Figure 10a. The charge transfer from the N1-nitrogen of isatin azanions compared with neutral isatins results in the reduction of the –N1–C2 and –N1–C8 bond lengths. Consequently, the double bond character of the C2=O carbonyl is also partially reduced. This effect (although to a lesser extent) is also applied to the C3=O carbonyl directly conjugated with the p-electrons of the phenyl ring. Despite the isatin phenyl ring substitution/modification (**B**, **C** or **D**), the characteristic feature of anionic structures is the shortening of the C3–C9, C4–C9, and C2–N1, C8–N1, C6–C7 bonds.

The substitution at position 5 of the phenyl ring by a suitable electron-withdrawing group (NO_2_ or =N–) leads to further quinone structure stabilization (structures **B** and **D**, Figure 10a). All these results agree well with the experimental results from UV–Vis, FTIR, and NMR spectroscopy. Figure 10b shows the structures with the largest bond length changes between the isatins and their azanions for each of the listed bonds.

As seen from Figure 10a,b, the largest difference in C2–C3 bond elongation (between carbonyls) is exhibited in azanion **D**. However, this result does not correlate with the smallest calculated difference in elongation for the azanion **B**. Experimentally measured and calculated UV–Vis spectra (Supplementary Figure S14) are in good agreement. Absorption bands (Table 6) in calculated UV–Vis spectra are hypsochromically shifted compared with the experimentally obtained ones. A good correlation was also found in the relative intensity of the bands and their shape (Supplementary Figure S14).

## 3. Materials and Methods

### 3.1. General Information

All chemicals were purchased from Sigma-Aldrich (St. Louis, MO, USA). Solvents were dried and purified by standard methods prior to use. Attenuated total reflection Fourier transform infrared (ATR-FTIR) spectra of all the described experiments were measured on a Nicolet 6700 FTIR (from ThermoNicolet Corp., Madison, WI, USA), using an ATR device with a diamond crystal plate. Spectra for liquid samples were measured in a cell with CaF_2_ windows and a path length of 0.2 mm. Spectra were recorded with ATR mathematical corrections yielding a 1.0 cm^−1^ actual resolution and 40 measurements were averaged. NMR spectra were recorded in 5 mm NMR tubes on a Varian VNMRS 600 MHz spectrometer (600 MHz for ^1^H; 150 MHz for ^13^C, Varian, Inc., Palo Alto, CA, USA) in DMSO-d_6_. Chemical shifts are referenced to tetramethylsilane (TMS) as an internal standard. Flash chromatography was performed on Merck (Darmstadt, Germany) silica gel 60. Thin-layer chromatography (TLC) was performed on Merck (Darmstadt, Germany) TLC-plate silica gel 60 F_254_. Chromatography was used for purification of prepared compounds (**C** and **E**). The elemental analysis was obtained using a Vario Micro cube Elementar CHNS analyzer (Elementar Analysensysteme GmbH, Hanau, Germany). Indoline-2,3-dione (**A**), 5-nitroindoline-2,3-dione (**B**) were purchased from Sigma-Aldrich (St. Louis, MO, USA), 4,6-dimethoxyindoline-2,3-dione (**C**) and 4-phenylindoline-2,3-dione (**E**) were synthesized according to references [73,74]. The p*K*_a_ values of the studied compounds **A**–**E** were determined spectrophotometrically by UV–Vis spectroscopy (Agilent 8453, cell 1 cm, resolution 1 nm, Santa Clara, California). Titration was performed in a DMSO/buffer solution at ratio 9:1 *v*/*v* in room temperature. Britton–Robinson universal buffer was used for titration. The pH of the solutions was measured by pH meter WTW (Weilheim, Germany) inoLab 720.

### 3.2. Synthesis

#### 3.2.1. Synthesis of Compound **D**

A mixture of 5-amino-1,10-phenanthroline (500 mg, 2.6 mmol) and diethyl ketomalonate (0.44 mL, 2.86 mmol) in glacial acetic acid (20 mL) was heated to reflux for 8 h. After cooling, the solvent was distilled off and a 5% solution of NaOH (50 mL) was added to the residue. The resulting solution was stirred at room temperature for 16 h. The mixture was treated with concentrated hydrochloric acid to achieve an acidic pH. The precipitated sold was filtered off, washed with water (50 mL), and dried.

*5H-pyrrolo[3,2-f][1,10]phenanthroline-6,7-dione* (**D**). Yield 65%; orange solid, m.p.: >350 °C. ^1^H-NMR (600 MHz, DMSO): δ 12.21 (s, 1H), 9.38 (d, *J* = 3.2 Hz, 1H), 9.13 (d, *J* = 8.2 Hz, 1H), 9.02 (d, *J* = 3.6 Hz, 1H), 8.94 (d, *J* = 8.2 Hz, 1H), 8.06 (ddd, *J* = 15.6, 8.3, 4.6 Hz, 2H). ^13^C-NMR (150 MHz, DMSO): δ 182.16; 160.90; 155.9; 154.79; 146.58; 146.15; 138.28; 134.84; 134.00; 126.72; 125.72; 124.88; 118.76; 104.76. Anal. calcd. for C_14_H_7_N_3_O_2_: C 67.47; H 2.83; N 16.86; found: C: 67.50; H 2.85; N 16.84.

#### 3.2.2. General Preparation of Isatin Azanions

Preparation of the isatin azanions was carried out by the concentration of isatins (1 × 10^−4^ mol·dm^−3^) with corresponding solutions of tetrabutylammonium salts (F^−^, AcO^−^) at the concentration of 1 × 10^−3^ mol·dm^−3^ in DMSO, DMF, and CH_3_CN.

### 3.3. Quantum-Chemical Calculations

The theoretical molecular structure and UV–Vis spectra of the isatin derivatives have been investigated by use of quantum-chemical calculations. The geometries of the structures were optimized at the M062X 6-31+g(dp) level. Stationary points were characterized as minima by computations of harmonic vibrational frequencies at the same theory level as geometry optimization. UV–Vis spectra and single point energies have been further calculated at time-dependent M062X levels of theory in combination with the 6-311++G(dp) basis set including the integral equation formalism-polarizable continuum model (IEF-PCM) solvation model. The zero-point vibrational energies and thermal corrections to the free energies were determined by using the unscaled M062X 6-31+g(dp) frequencies.

## 4. Conclusions

Five isatin azanions **A**–**E** were prepared in an aprotic environment. Different basic anions—F^−^ (TBAF), OH^−^ (TBAOH), or acetate (TBAAc) ions (with different counter ion)—were used for the amide NH group deprotonation. The azanions’ stability strongly depends on the base used for deprotonation. The usage of F^−^ and CH_3_COO^−^ anions results in the formation of isatin azanions which are stable at laboratory temperature even at a 200-fold excess of base. However, a higher OH^−^ concentration than 1 equiv of isatin leads to the nucleophilic attack of the isatin C-2 carbon, followed by the isatin five-membered ring opening to the corresponding 2-(2-aminophenyl)-2-oxoacetic acid. Prepared azanions were characterized by UV–Vis, FTIR, and NMR spectroscopy. Depending on the azanion structure, the nitrogen free electron pair formed by the deprotonation is in less or more conjugation with the phenyl ring of the azanions. Interestingly, aggregation occurs at azanion concentration higher than 1 × 10^−3^ mol·dm^−3^. The process of the **A**–**E** azanion formation is reversible. In the presence of proton donor solvents, the prepared azanions change to the initial isatins. Counter ion parameters affect the UV–Vis and FTIR spectra of the prepared azanions. We assume that the differences in spectra result from different bonding properties between the cation and corresponding azanion. The alkali metal, TBA^+^, and Mg^2+^ cations do not have a free electron pair in the valence sphere. They have the characteristic of hard acids and, therefore, their binding to the azanion has predominantly coulombic character. Due to the large shift of the C-2 *ν(C=O)* vibrational to the lower wavelength in azanions, we assume that these cations interact predominantly with a negative charge localized on the C-2 carbonyl oxygen of isatin. Contary to these cations, the Cd^2+^, Zn^2+^, Hg^2+^, Co^2+^, and Cu^+^ counter ions interact with isatin nitrogen. The FTIR spectra confirm that even the Ag^+^ cation interacts predominantly with the C-2 isatin carbonyl oxygen. Azanions with different cations can be prepared directly from the starting isatins (**A**–**E**) using either the appropriate acetate or fluoride salts, although the weak solubility of the fluoride salts strongly restricts the isatin azanion’s preparation with these cations. However, isatin azanions with different cations can be prepared also from the isatin TBA^+^ salts by TBA^+^ exchange for the corresponding cation.

## Figures and Tables

**Table 1:** UV–Vis spectra of **A**–**E** and their azanions (1 × 10^−4^ mol·dm^−3^) in DMSO and CH_3_CN.

Compound	DMSO				CH_3_CN			
	λ_max_/logε	λ_max_/logε	λ_max_/logε	λ_max_/logε	λ_max_/logε	λ_max_/logε	λ_max_/logε	λ_max_/logε
**A**	259/3.43	298/3.53	415/2.93	-	242/4.37	295/3.55	408/2.91	-
**A**_azanion_	266/4.30	297P/3.46	-	569/2.82	255/4.41	292P/3.41	-	555/2.75
**B**	278/3.93	329/3.89	-	-	275/4.24	316/4.02	-	-
**B**_azanion_	294P/3.63	296P/3.61	417/4.20	531P/3.34	290P/3.74	-	410/4.36	508/3.48
**C**	-	348/3.92	409P/3.11	-	-	343/3.92	400P/3.11	-
**C**_azanion_	269/4.43	340/3.72	-	509/2.89	265/4.41	338/3.67	-	512/2.82
**D**	-	309/3.89	461/3.38	-	278P/3.56	320P/3.12	443/2.90	-
**D**_azanion_ *	277/4.46	322/3.99	421/3.08	610/3.24	274/3.86	320/3.35	405/2.47	597/2.60
**E**	-	329/3.52	421/3.22	-	-	327/3.41	416/3.18	-
**E**_azanion_	290P/4.01	341P/3.26	-	576/2.95	284P/3.98	331P/3.22	-	554/2.90

* Saturated solution**,** P: Shoulder. λ_max_: maximum absorption, ε: molar attenuation coefficient.

**Table 2:** Rate constants to characterize VI or V (**A**–**E**) formation (1 × 10^−4^ mol·dm^−3^) in the presence of tetrabutylammonium hydroxide (TBAOH) (1 × 10^−2^ mol·dm^−3^) in DMSO.

DMSO	10^−2^ *k_TBAOH_* (s^−1^)
**B** **	5.98
**E**	2.84
**D**	1.77
**A**	1.34
**C**	0

Calculation of *k_TBAOH_* from loss of azanion absorbance (formation of **V**). ** Calculation of *k_TBAOH_* from the increase in the open-form absorbance (formation of **VI**).

**Table 3:** Position of the absorption maxima **A**–**E** isatin derivative carbonyls (*ν_s_(C=O)*, *ν_as_(C=O))* and their azanions (**A**–**E**).

Compound *		ATR/(cm^−1^)	CH_3_CN/(cm^−1^)	CHCl_3_/(cm^−1^)
**A**	*ν_s_(C=O)*	1724	1744	1743
	*ν_as_(C=O)*	1746	1761	1760
**A**_azanion/TBAF_	*ν_s_(C=O)*	1716	1720	1719
	*ν_as_(C=O)*	1645	1632	1645
**A**_azanion/Ag_^+^	*ν_s_(C=O)*	1723	-	-
	*ν_as_(C=O)*	1570	-	-
**A**_azanion/Hg_^2+^	*ν_s_(C=O)*	1733	-	-
	*ν_as_(C=O)*	1676	-	-
**B**	*ν_s_(C=O)*	1749, 1732	1757	1754
	*ν_as_(C=O)*	1770	1768	1774p
**B**_azanion/TBAF_	*ν_s_(C=O)*	1735	1735	1734
	*ν_as_(C=O)*	1674	1677	1672
**B**_azanion/Ag_^+^	*ν_s_(C=O)*	1743	-	-
	*ν_as_(C=O)*	1654, 1597	-	-
**B**_azanion/Hg_^2+^	*ν_s_(C=O)*	1745p,1734	-	-
	*ν_as_(C=O)*	1696, 1605	-	-
**C**	*ν_s_(C=O)*	1730	-	-
	*ν_as_(C=O)*	1748	-	-
**C**_azanion/TBAF_	*ν_s_(C=O)*	1716	-	-
	*ν_as_(C=O)*	1646	-	-
**C**_azanion/Ag_^+^	*ν_s_(C=O)*	1714	-	-
	*ν_as_(C=O)*	1634	-	-
**D**	*ν_s_(C=O)*	1732	-	-
	*ν_as_(C=O)*	1763	-	-
**D**_azanion/TBAF_	*ν_s_(C=O)*	1681	-	-
	*ν_as_(C=O)*	1650p	-	-
**D**_azanion/Ag_^+^	*ν_s_(C=O)*	1705	-	-
	*ν_as_(C=O)*	1685p	-	-
**E**	*ν_s_(C=O)*	1731	-	-
	*ν_as_(C=O)*	1743	-	-
**E**_azanion/TBAF_	*ν_s_(C=O)*	1727	-	-
	*ν_as_(C=O)*	1652	-	-
**E**_azanion/Ag_^+^	*ν_s_(C=O)*	1710	-	-
	*ν_as_(C=O)*	1634	-	-

Attenuated total reflection (ATR); * Vibrational modes: ν-Stretching.

**Table 4:** Chemical shifts of the **A**–**E** isatin derivatives and their azanions in ^1^H-NMR in DMSO.

Compound/Hydrogen	A (ppm)	A_azanion_ (ppm)	B (ppm)	B_azanion_ (ppm)	C (ppm)	C_azanion_ (ppm)	D (ppm)	D_azanion_ (ppm)	E (ppm)	E_azanion_ (ppm)
H1	11.03	-	11.63	-	10.94	-	12.25	-	11.10	-
H4	7.48	7.11	8.20	7.71	-	-	-	-	-	-
H5	7.05	6.64	-	-	6.15	6.06	-	-	6.99	6.29
H6	7.57	7.27	8.43	8.04	-	-	-	-	7.58	7.11
H7	6.89	6.62	7.06	6.55	5.98	6.06	-	-	6.87	6.36
	-	-	-	-	3.85	3.83	-	-	-	-
	-	-	-	-	3.83	3.82	-	-	-	-
H10	-	-	-	-	-	-	9.14	8.45	-	-
H11	-	-	-	-	-	-	8.04	7.43	7.53	7.40
H12	-	-	-	-	-	-	9.03	8.49	-	-
H15	-	-	-	-	-	-	9.38	9.06	-	-
H16	-	-	-	-	-	-	8.09	7.65	-	-
H17	-	-	-	-	-	-	8.97	8.64	-	-
H18	-	-	-	-	-	-	-	-	7.43	7.35
H19	-	-	-	-	-	-		-	7.43	7.30
H20	-	-	-	-	-	-	-	-	7.43	7.35
H21	-	-	-	-	-	-	-	-	7.53	7.40

**Table 5:** Chemical shifts of the **A**–**E** isatin derivatives and their azanions in ^13^C-NMR in DMSO.

Compound/Carbon	A (ppm)	A_azanion_ (ppm)	B (ppm)	B_azanion_ (ppm)	C (ppm)	C_azanion_ (ppm)	D (ppm)	D_azanion_ (ppm)	E (ppm)	E_azanion_ (ppm)
C2	159.79	166.16	160.32	172.76	162.00	162.87	160.90	175.05	159.46	170.67
C3	184.81	192.92	182.80	194.93	178.86	179.88	182.16	193.00	183.40	197.10
C4	125.12	123.86	120.02	119.07	160.71	160.55	124.88	126.94	141.94	140.13
C5	123.19	119.76	143.05	137.92	92.44	92.08–92.19	138.28	140.75	124.76	118.93
C6	138.80	138.30	133.50	134.08	170.17	170.13	146.58	151.16	138.31	137.52
C7	112.63	114.65	112.94	115.88	91.95	92.08–92.19	118.76	124.48	111.52	115.48
C8	151.15	164.16	155.62	178.84	155.08	156.93	155.90	182.50	151.90	174.99
C9	118.26	119.65	118.59	119.34	100.97	101.08	104.76	102.42	114.65	116.87
C4-OCH_3_	-	-	-	-	57.98	57.96	-	-	-	-
C6-OCH_3_	-	-	-	-	56.69	56.33	-	-	-	-
C10	-	-	-	-	-	-	134.84	127.89	136.77	138.32
C11	-	-	-	-	-	-	126.72	124.65	129.28	128.91
C12	-	-	-	-	-	-	146.15	144.23	-	-
C13	-	-	-	-	-	-	-	-	-	-
C14	-	-	-	-	-	-	-	-	-	-
C15	-	-	-	-	-	-	154.79	153.58	-	-
C16	-	-	-	-	-	-	125.72	123.23	-	-
C17	-	-	-	-	-	-	134.00	133.04	-	-
C18	-	-	-	-	-	-	-	-	128.49	128.09
C19	-	-	-	-	-	-	-	-	129.06	128.09
C20	-	-	-	-	-	-	-	-	128.49	128.09
C21	-	-	-	-	-	-	-	-	129.28	128.91

**Table 6:** Calculated values of deprotonation energies for isatin derivatives **A**–**E**, absorption maxima values from calculated UV–Vis spectra, and experimentally determined p*K*_a_ values.

	∆*E* (kJ·mol^−1^) *	p*K*_a_	λ_max_	λ_max(azanion)_
**A**	1206.7	8.59	374; 261; 218	488; 237
**B**	1181.1	6.38	357; 256; 211	461; 344; 231
**C**	1213.1	8.67	312; 213	446; 297; 241
**D**	1196.0	6.65	420; 240	529; 352p; 261
**E**	1205.6	8.57	376; 312; 229	500; 266; 234

* ∆*E*: overall Gibb’s free energy change after deprotonation; ∆*E* (kJ·mol^−1^) = **E**_molecule_ − **E**_azanion_.

## Supplementary Materials

Supplementary Materials are available online.
