# Supplementary material for: Effect of Structure on Charge Distribution in the Isatin Anions in Aprotic Environment: Spectral Study
Source: Molecules. 2017 Nov 14;22(11):1961. doi: 10.3390/molecules22111961 (PMC6150331; doi:10.3390/molecules22111961)
Supplement: Supplementary file 1 [file molecules-22-01961-s001.pdf]

Effect of structure on charge distribution in the isatin anions in aprotic environment: spectral study.

Pavol Tisovský [1*], Róbert Šandrik [1], Miroslav Horváth [1], Jana Donovalová [1], Juraj Filo [1], Martin Gáplovský [2], Klaudia Jakusová [1], Marek Cigáň [1], Robert Sokolík [1], Anton Gáplovský

[1] *Faculty of Natural Sciences, Institute of Chemistry, Comenius University, Ilkovičova 6, Mlynská dolina CH-2, SK-842 15 Bratislava, Slovakia; pavol.tisovsky@uniba.sk (P.T.); sandrik2@uniba.sk (R.Š.); mirek.horvath@gmail.com (M.H.); donovalova@fns.uniba.sk (J.D.); filo@fns.uniba.sk (J.F.); jakusova@fns.uniba.sk (K.J.); cigan@fns.uniba.sk (M.C.); sokolik@fns.uniba.sk (R.S.) ; gaplovsky@fns.uniba.sk (A.G.)*

[2] *Department of Pharmaceutical Chemistry, Faculty of Pharmacy, Comenius University, Odbojárov 10, SK-832 32 Bratislava, Slovakia; m.gaplovsky@yahoo.com*

[*] *Correspondence: pavol.tisovsky@uniba.sk; Tel.: +421-2-60296378*

*pavol.tisovsky@uniba.sk

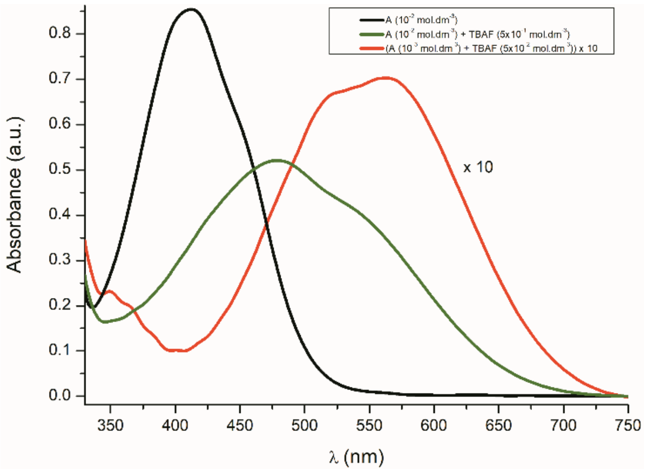

Figure S1. Concentration effect of the **A**-azanion on the UV-Vis spectra in CH_3CN.

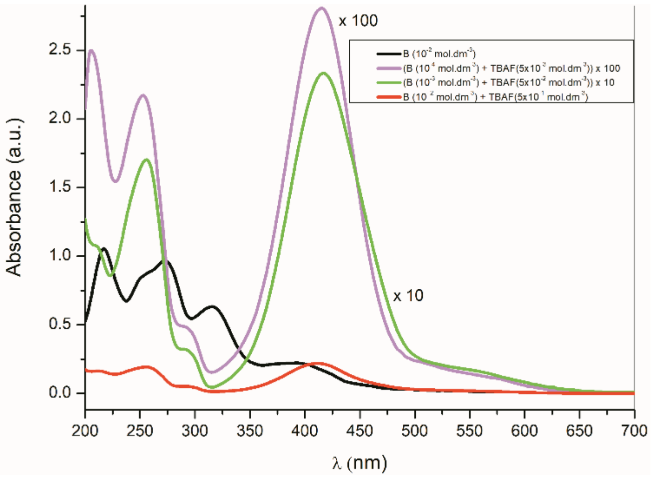

Figure S2. UV-Vis spectral changes of **B**-azanion depending on concentration of **B** in the presence of TBAF in CH_3CN.

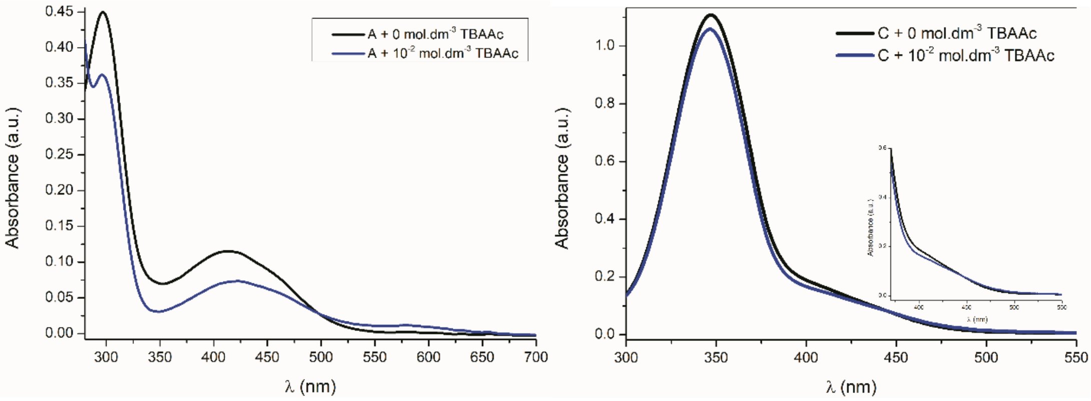

Figure S3. Change in UV-Vis spectra **A** and **C** (1×10^{-4} mol.dm^{-3}) after addition of TBAAc in DMSO.

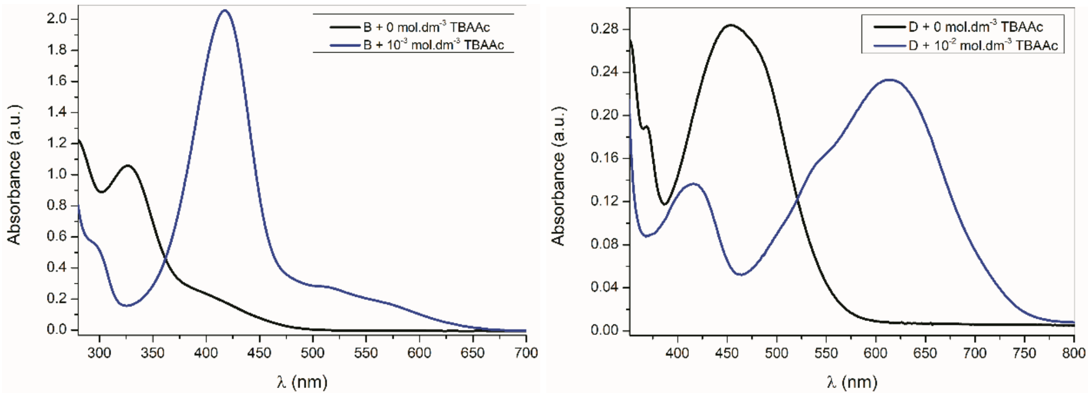

Figure S4. Effect of TBAAc on UV-Vis spectra of **B** and **D** (1×10^{-4} mol.dm^{-3}) in DMSO.

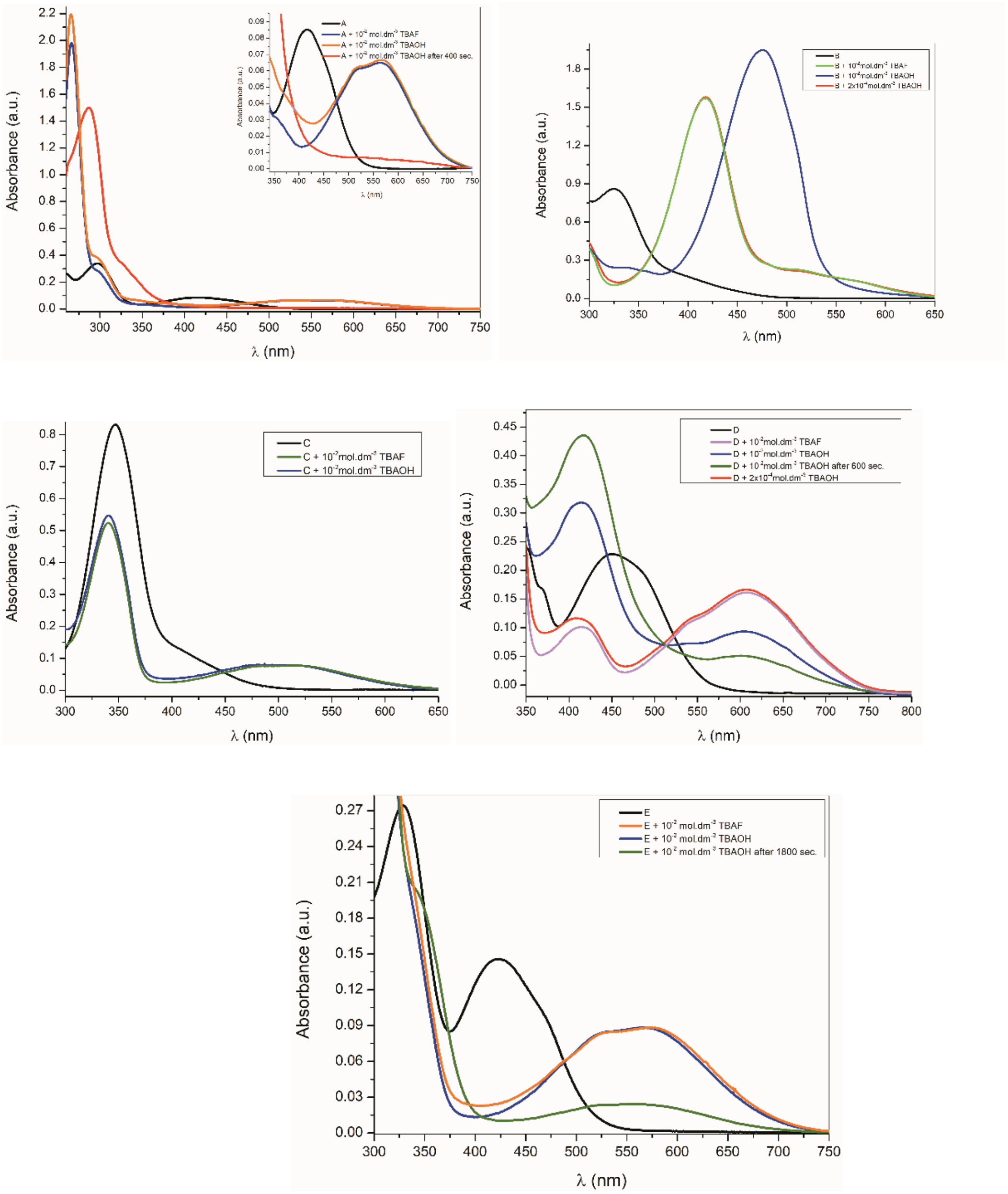

Figure S5. Change of UV-Vis spectra of **A** - **E** (1×10^{-4} mol.dm^{-3}) in the presence of TBAF and TBAOH in DMSO.

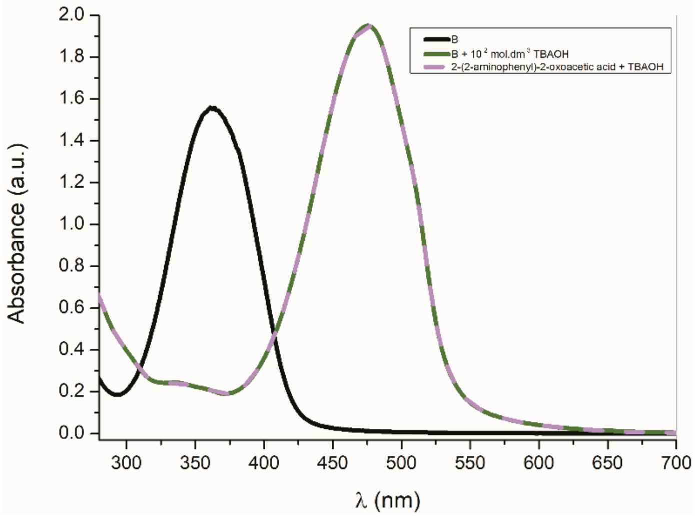

Figure S6. UV-Vis spectrum of TBA salt 2-(2-aminophenyl)-2-oxoacetic acid (1×10^{-4} mol.dm^{-3}) and UV-Vis spectrum of **B** (1×10^{-4} mol.dm^{-3}) after addition TBAOH in DMSO.

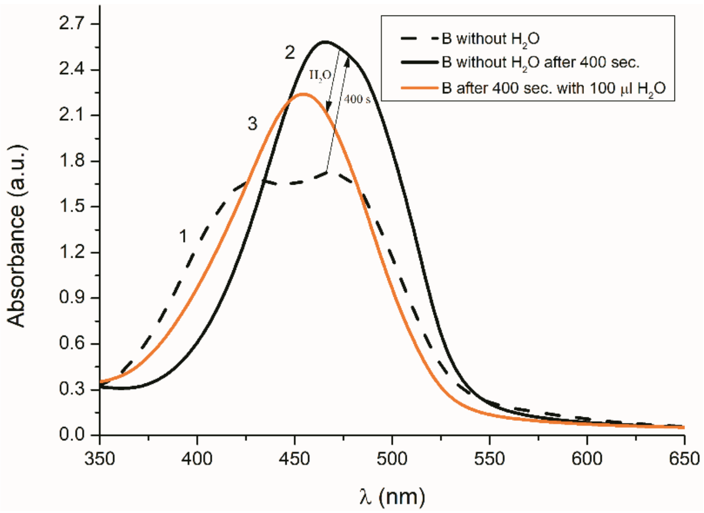

Figure S7. Effect of water on **B** UV-Vis spectra (1×10^{-4} mol.dm^{-3}) in the presence of TBAOH (1×10^{-2} mol.dm^{-3}) in DMSO.

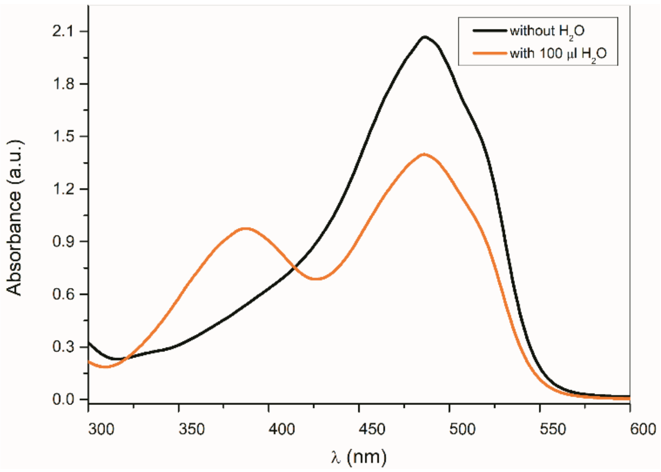

Figure S8. Effect of water on UV-Vis spectra 1-metyl-5-nitroisatin (1×10^{-4} mol.dm^{-3}) in the presence of TBAOH (1×10^{-2} mol.dm^{-3}) in DMSO.

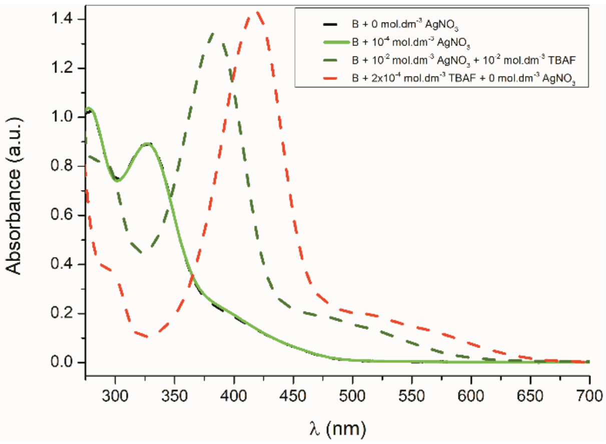

Figure S9. $AgNO_3$ effect on UV-Vis spectra of **B** (1×10^{-4} mol.dm^{-3}) in DMSO.

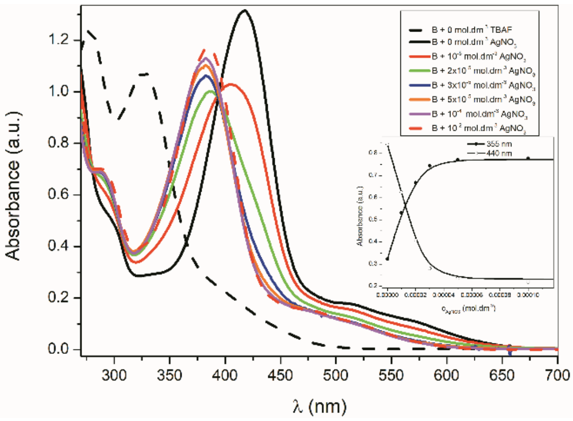

Figure S10. **B** UV-Vis spectra change (1×10^{-4} mol.dm^{-3}) with TBAF (1×10^{-2} mol.dm^{-3}) depending on $AgNO_3$ concentration in DMSO.

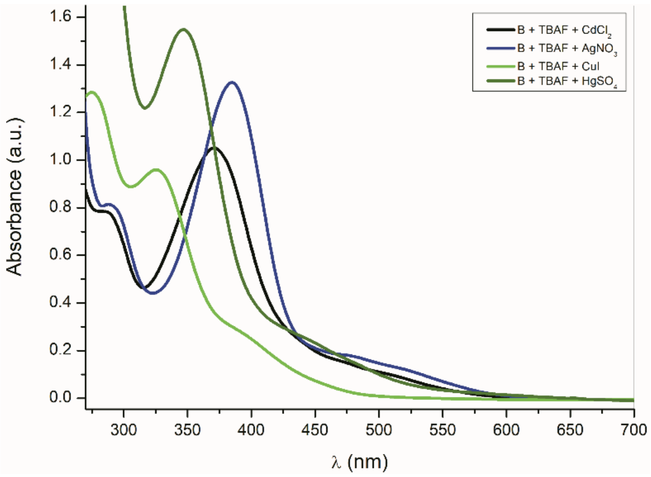

Figure S11. **B** UV-Vis spectra in DMSO in the presence of TBAF (1×10^{-3} mol.dm^{-3}) after $CdCl_2$, $AgNO_3$, CuI and $HgSO_4$ addition.

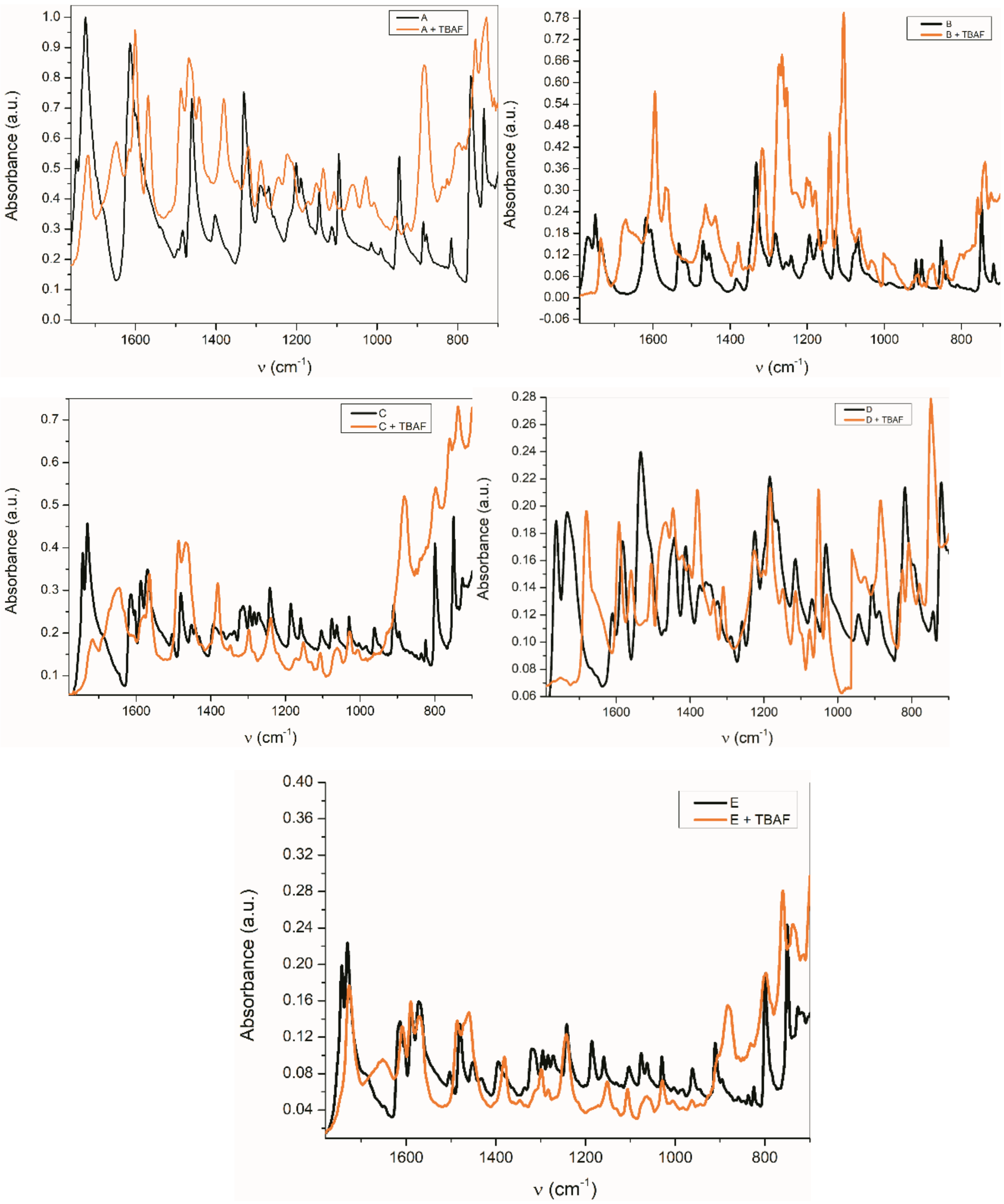

Figure S12. FTIR spectra (ATR) **A - E** and their azanions

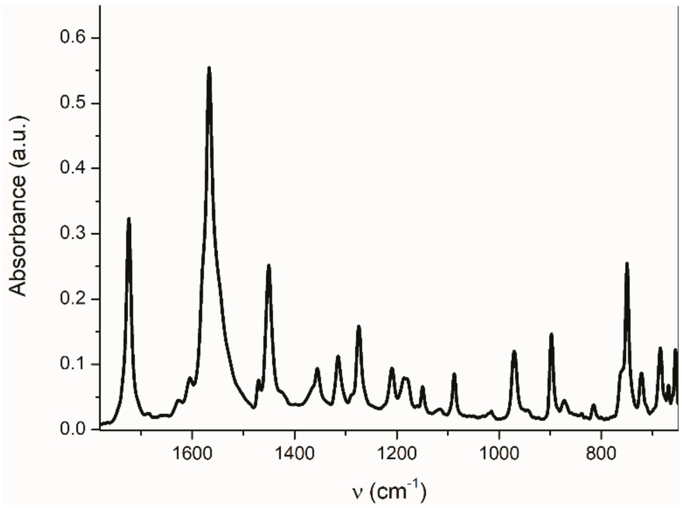

Figure S13. FTIR spectrum of the silver salt of **A** (ATR spectrum).

Table S1. Calculated bond lengths (Å) of structures **A - E** and of their azanions.

	A	$A_{azanion}$	B	$B_{azanion}$	C	$C_{azanion}$	D	$D_{azanion}$	E	$E_{azanion}$
N_1-H	1.00763	-	1.00834	-	1.00913	-	1.00819	-	1.00947	-
C_2-N_1	1.37969	1.35211	1.38667	1.36918	1.38284	1.35777	1.38617	1.37576	1.37717	1.35345
C_2-O	1.19550	1.21998	1.19223	1.21115	1.20308	1.22498	1.20073	1.21691	1.20324	1.22528
C_2-C_3	1.56764	1.58531	1.56581	1.57919	1.56890	1.58737	1.56901	1.59239	1.56607	1.58466
C_3-O	1.19476	1.20623	1.19252	1.20285	1.20477	1.21400	1.20607	1.21988	1.20302	1.21352
C_3-C_9	1.47322	1.46741	1.47680	1.46826	1.46235	1.46096	1.46545	1.44472	1.48125	1.47533
C_9-C_4	1.38283	1.37975	1.37793	1.36787	1.39744	1.38759	1.43228	1.42157	1.40112	1.39909
C_4-O	-	-	-	-	1.33998	1.36270	-	-	-	-
O-CH_3	-	-	-	-	1.41678	1.40464	-	-	-	-
C_4-C_5	1.38992	1.39311	1.38637	1.39795	1.40411	1.40506	1.41481	1.42356	1.40381	1.40228
C_5-N	-	-	1.46902	1.43231	-	-	-	-	-	-
N-O	-	-	1.21222	1.22580	-	-	-	-	-	-
N-O	-	-	1.21330	1.22747	-	-	-	-	-	-
C_5-C_6	1.39246	1.39998	1.38936	1.40682	1.40103	1.40960	1.46719	1.47271	1.39290	1.40307
C_6-O	-	-	-	-	1.34937	1.37430	-	-	-	-
O-CH_3	-	-	-	-	1.41623	1.40460	-	-	-	-
C_6-C_7	1.39370	1.38722	1.38770	1.37285	1.40632	1.38868	1.42081	1.41731	1.39711	1.38717
C_7-C_8	1.38173	1.40583	1.38570	1.41722	1.37739	1.40552	1.42976	1.45425	1.38474	1.41092
C_8-C_9	1.39827	1.41907	1.40224	1.43186	1.40875	1.43192	1.37219	1.40523	1.40812	1.43027
C_8-N_1	1.40099	1.37003	1.39082	1.34657	1.39688	1.36400	1.39677	1.34718	1.40115	1.36753
C_4-C_{10}	-	-	-	-	-	-	1.40937	1.41323	1.48382	1.48756
C_{10}-C_{11}	-	-	-	-	-	-	1.37714	1.37882	1.40025	1.40107
C_{11}-C_{12}	-	-	-	-	-	-	1.40886	1.40654	-	-
C_{12}-N_{13}	-	-	-	-	-	-	1.31974	1.32345	-	-
N_{13}-C_5	-	-	-	-	-	-	1.34576	1.34424	-	-
C_6-N_{14}	-	-	-	-	-	-	1.34651	1.34965	-	-

**N_{14}-C_{15}**	-	-	-	-	-	-	1.31934	1.32276	-	-
**C_{15}-C_{16}**	-	-	-	-	-	-	1.40900	1.40436	-	-
**C_{16}-C_{17}**	-	-	-	-	-	-	1.37510	1.38096	-	-
**C_{17}-C_{7}**	-	-	-	-	-	-	1.40817	1.40101		
**C_{11}-C_{18}**	-	-	-	-	-	-	-	-	1.39202	1.39330
**C_{18}-C_{19}**	-	-	-	-	-	-	-	-	1.39357	1.39375
**C_{19}-C_{20}**	-	-	-	-	-	-	-	-	1.39429	1.39496
**C_{20}-C_{21}**	-	-	-	-	-	-	-	-	1.39103	1.39165
**C_{21}-C_{10}**	-	-	-	-	-	-	-	-	1.39897	1.40039

Table S2. Calculated charge densities of atoms in structures **A** - **E** and of their azanions.

	**A**	**$A_{azanion}$**	**B**	**$B_{azanion}$**	**C**	**$C_{azanion}$**<br>-	**D**	**$D_{azanion}$**	**E**	**$E_{azanion}$**
**N-H**	0.318	-	0.327		0.321	-	0.300	-	0.320	-
**N_{1}**	-0.335	-0.581	-0.321	-0.528	-0.368	-0.585	-0.313	-0.589	-0.314	-0.563
**C_{2}**	0.440	0.516	0.442	0.515	0.391	0.552	0.375	0.428	0.433	0.546
**O**-C_{2}	-0.491	-0.622	-0.467	-0.569	-0.498	-0.516	-0.461	-0.560	-0.497	-0.622
**C_{3}**	0.180	-0.031	0.335	0.161	0.312	-0.030	0.388	0.316	-0.014	-0.188
**O**-C_{3}	-0.450	-0.509	-0.436	-0.489	-0.495	-0.545	-0.469	-0.551	-0.429	-0.491
**C_{4}**	-0.368	-0.213	-0.636	-0.436	0.541	0.327	1.099	1.059	0.555	0.543
**C_{4}-O**	-	-	-	-	-0.325	-0.346	-	-	-	-
**O-CH_{3}**	-	-	-	-	-0.307	-0.345	-	-	-	-
**C_{5}**	-0.107	-0.162	0.210	0.147	0.47	0.428	-0.678	-0.760	-0.384	-0.416
**C_{6}**	-0.153	-0.164	-0.013	-0.003	-0.161	-0.127	-0.516	-0.491	0.264	-0.288
**C_{6}-O**	-	-	-	-	-0.306	-0.335	-	-	-	-
**O-CH_{3}**	-	-	-	-	-0.307	-0.295	-	-	-	-
**C_{7}**	-0.348	-0.154	-0.591	-0.388	-0.384	-0.859	1.151	0.856	-0.699	-0.530
**C_{8}**	-0.516	-0.617	-0.485	-0.604	-0.467	-0.418	0.028	0.129	-0.274	-0.365
**C_{9}**	1.190	0.964	1.380	1.108	0.153	0.800	0.119	-0.084	1.23	1.102
**C_{10}**	-	-	-	-	-	-	-0.624	-0.584	0.837	0.784
**C_{11}**	-	-	-	-	-	-	-0.435	-0.421	-0.430	-0.401
**C_{12}**	-	-	-	-	-	-	-0.006	-0.029	-	-
**N_{13}**	-	-	-	-	-	-	-0.232	-0.255	-	-
**N_{14}**	-	-	-	-	-	-	-0.205	-0.239	-	-
**C_{15}**	-	-	-	-	-	-	0.099	0.094	-	-
**C_{16}**	-	-	-	-	-	-	-0.234	-0.368	-	-
**C_{17}**	-	-	-	-	-	-	-0.371	0.090	-	-
**C_{18}**	-	-	-	-	-	-	-	-	-0.230	-0.222
**C_{19}**	-	-	-	-	-	-	-	-	-0.229	-0.248
**C_{20}**	-	-	-	-	-	-	-	-	-0.251	-0.247
**C_{21}**	-	-	-	-	-	-	-	-	-0.609	-0.570

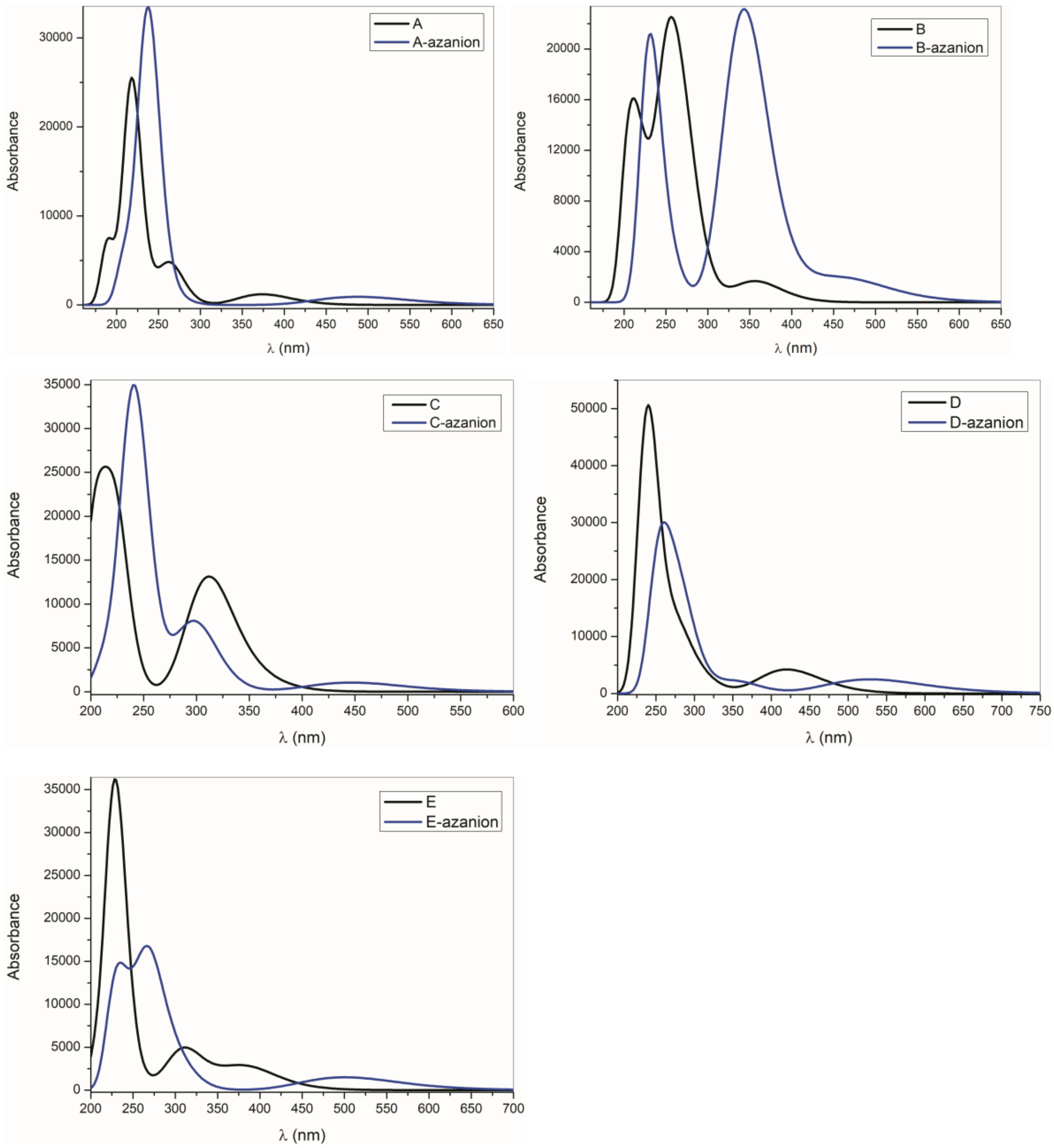

Figure S14. Calculated UV-Vis spectra of isatin derivatives (**A-E**) and their respective azanions.
